# Sociocultural Context of Suicidal Behaviour in the Sundarban Region of India

**DOI:** 10.1155/2013/486081

**Published:** 2013-06-11

**Authors:** A. N. Chowdhury, S. Banerjee, A. Brahma, A. Hazra, M. G. Weiss

**Affiliations:** ^1^Institute of Psychiatry, 7 D. L. Khan Road, Kolkata 700025, India; ^2^Northamptonshire Healthcare NHS Foundation Trust, Stuart Road Resource Centre, Corby, Northants NN17 1RJ, UK; ^3^Department of Pharmacology, IPGME & R, 244 A.J.C. Bose Road, Kolkata 700020, India; ^4^Department of Epidemiology & Public Health, Swiss Tropical & Public Health Institute, Socinstrasse 57, 4051 Basel, Switzerland

## Abstract

The role of mental illness in nonfatal deliberate self-harm (DSH) is controversial, especially in Asian countries. This prospective study examined the role of psychiatric disorders, underlying social and situational problems, and triggers of DSH in a sample of 89 patients hospitalised in primary care hospitals of the Sundarban Delta, India. Data were collected by using a specially designed DSH register, Explanatory Model Interview Catalogue (EMIC), and clinical interview. Psychiatric diagnosis was made following the DSM-IV guidelines. The majority of subjects were young females (74.2%) and married (65.2%). Most of them (69.7%) were uncertain about their “intention to die,” and pesticide poisoning was the commonest method (95.5%). Significant male-female differences were found with respect to education level, occupation, and venue of the DSH attempt. Typical stressors were conflict with spouse, guardians, or in-laws, extramarital affairs, chronic physical illness, and failed love affairs. The major depressive disorder (14.6%) was the commonest psychiatric diagnosis followed by adjustment disorder (6.7%); however 60.7% of the cases had no psychiatric illness. Stressful life situations coupled with easy access to lethal pesticides stood as the risk factor. The sociocultural dynamics behind suicidal behaviour and community-specific social stressors merit detailed assessment and timely psychosocial intervention. These findings will be helpful to design community-based mental health clinical services and community action in the region.

## 1. Introduction

Presently both fatal and nonfatal deliberate self-harm (DSH) are major public health concerns globally. Fatal DSH (i.e., suicidal death) accounted for more than 900,000 lives lost in 1995 [1]. The rate of non-fatal DSH is 10 times more than fatal DSH [2]. In India, the suicide rate is approximately 11.6 per 100,000 populations [3]. Research has revealed that young age, female sex, low educational level [4], being married [5], and housewife status [6] are potential risk factors for both fatal and non-fatal DSH. Several studies also document psychiatric illness, especially major depression, as associated with DSH behaviour [7, 8]. However, the relationship between suicidal behaviour and designated psychiatric illnesses is a matter of great controversy. Unlike some western studies [9–11] in which psychiatric and personality disorders are referred to as predominant DSH antecedents, some Indian urban studies [12, 13] have pointed out that many people who come to clinical attention after DSH do not have any designated Axis I (DSM IV) psychiatric disorder; rather they are stressed by psychosocial factors like adjustment problems or social and situational factors. Few recent western and other studies also highlighted the fact that mental health factors are “not the sole indicators of risk of self-harm” [14, 15]; on the contrary, the life events and psychosocial factors [16] play a significant role in DSH behaviours. Detailed psychiatric assessment of self-harm patients is always difficult, and especially so at the primary care level. There is no convincing data available till date to comment on the relation of designated mental illnesses with DSH at the primary care level. So this study was addressed to examine this relation at primary care. 

The Sundarban comprises a relatively remote and inaccessible coastal region in West Bengal and covers 19 community developmental blocks, 13 of which fall in the South 24 Parganas district and 6 under the North 24 Parganas district, West Bengal state of India. The present study is from the Sundarban blocks under the South 24 Parganas district. A previous study [17] involving retrospective analysis of 1850 DSH admission data of 1999 from all the 13 Block Primary Health Centres showed that a high proportion of attempters were female (65.5%) and poisoning was the commonest method (96.9%) of self-harm with 8.6% fatality. This study was thus designed to explore the clinical and demographic characteristics of non-fatal DSH cases with particular focus on gender distribution and intent to die among the attempters across the Sundarban region. There is also an attempt to examine whether designated psychiatric illnesses are associated with deliberate self-harm attempters admitted in the different Block Primary Health Centres (BPHC) of Sundarban delta during the year 2006.

**Figure 1:**
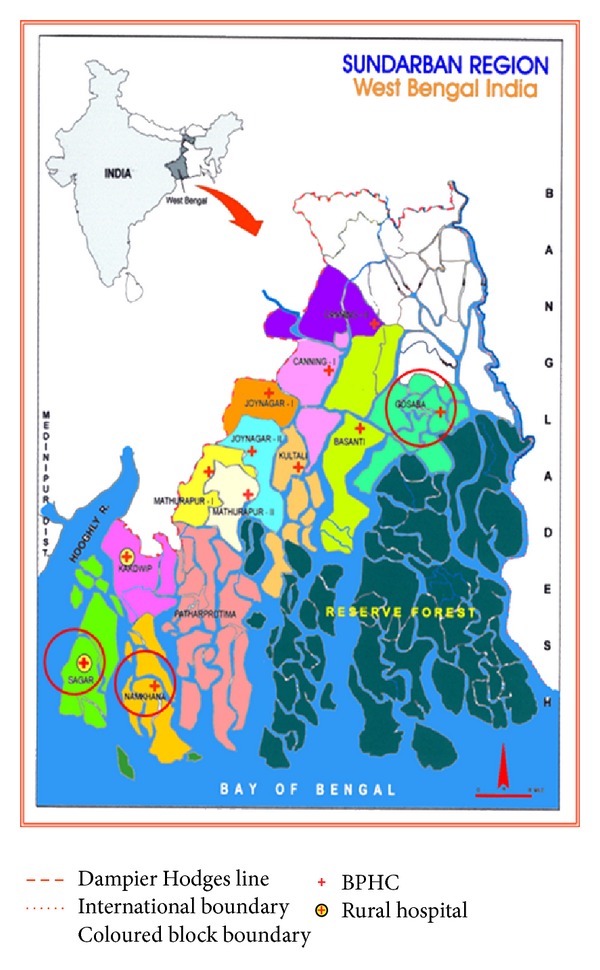
Sundarban region under the South 24 Parganas, showing the study blocks (not to scale).

*Study Area.* The Sundarban region is the largest delta in the world, at the confluence of the Ganges-Hooghly river in the Bay of Bengal. It spans the southernmost part of the state of West Bengal in India and the neighbouring regions of Bangladesh. There are 54 islands spread over an area of 1630 sq. km that is intersected by numerous canals and tidal creaks. Socioeconomically, the area is below the rest of the region (Human Development Index—0.55 in contrast to 0.62 of the district). Both the literacy rate and per capita income are much lower than the state average [18]. Out of 13 administrative blocks under the South 24 Parganas district, six blocks are island blocks and the rest are connected with the mainland. Sagar is the most western, Namkhana is in the middle, and the Gosaba is the most eastern island block of the Sundarban (Figure 1). Each block has one main clinical facility—the Block Primary Health Centre (BPHC). Sagar has an area of 282.11 sq. km, with a population of 1,85,644, Namkhana is 370.61 sq. km with a population of 1,60,627, and Gosaba is 296.73 sq. km with a population of 2,22,822 [19]. The study period and BPHCs were randomly selected as follows: February for Sagar, March for Namkhana, and April for Gosaba BPHC. In recent years pesticide poisoning and deliberate self-harm were recognised as an alarming public health problem in the Sundarban [20, 21]. The health service provision in the entire Sundarban region is poor and inadequate. There is no mental health service at all. This study was part of the broader research devoted to the development of a community-based mental health program in the region [22, 23] and thus attempted to provide the unmet clinical service needed in the region. Ethical clearance was granted by the office of the Chief Medical Officer of Health, South 24 Parganas district.

## 2. Materials and Methods 

### 2.1. Sample

All cases of DSH (non-fatal) admitted to the respective BPHCs in the study month, who gave written informed consent (or agreed by thumb impression to the study protocol read out to them) and completed the study protocol, were considered for analysis. A total of 89 cases (23 males and 66 females) consented and completed the interviews. The male : female distribution at each BPHC was as follows: Sagar 6 : 21, Namkhana 10 : 26, and Gosaba 7 : 19. 

### 2.2. DSH Register

A DSH register was designed for the collection of relevant demographic and clinical data for DSH cases. It included a 20-item case history sheet written in Bengali that was filled in during admission by the staff nurse or medical officer. The medical officer in charge and a designated staff nurse of each BPHC were trained for data recording in the DSH register. The response to “intention to die” (yes/no/uncertain) is recorded from the patient after stabilisation, by the staff nurse or medical officer. In Sundarban region the patient is usually accompanied by family members, for instance, spouse in married cases and parents in unmarried cases along with other solicitous relatives. Reporting of the “The cause of DSH” at initial screening was elicited from a responsible family member or relative present during the admission. Patient's opinion about the cause of DSH was elicited afterwards in EMIC interview.

### 2.3. Clinical Assessment and Instruments

The initial identification of cases as DSH was done by the block medical officer, and usually after 3-4 days of admission when the concerned medical officer declared the subject is fit enough, a detailed clinical interview was carried out. A written-informed consent was taken from the subject and the following steps were followed. As the total interview process was a lengthy one, it was divided into two sessions.

#### 2.3.1. Session 1: Diagnostic Interview

The following assessments were made: (a) screening for personality disorder (borderline type) by Self-Harm Inventory (SHI) [24] and Personality Diagnostic Questionnaire 4th edition (PDQ-4; consistent with DSM-IV diagnostic criteria for personality disorder) [25] and (b) psychiatric diagnosis by SCID module [26]. Psychiatric assessment was made jointly by a consultant (professor) and trainee psychiatrist.

#### 2.3.2. Session 2: EMIC Interview

The Explanatory Model Interview Catalogue [27] is an extended interview schedule in local language that is specially prepared for this purpose and intended for cultural epidemiological assessment (sociocultural perspective of underlying distress, meaning and experience of distress, and help seeking). It reveals the subject's own perspective, in his/her verbatim about the illness experience, its causes, and help seeking behaviour. EMIC interviews were conducted by a Ph.D. trainee and consultant psychiatrist.

### 2.4. Statistical Analysis

The data has been summarised by simple descriptive statistics. Fisher's exact test was applied to categorical variables and for normally distributed numerical variables, independent sample *t* test was used to assess the difference between groups. All analyses were 2-tailed, and *P* < 0.05 was considered statistically significant.

## 3. Results

### 3.1. Demographic Profile

In the study population, 21.7% of men were single and the rest were married; among the females, 18.2% were single, 78.8% were married, and 3% were widowed. Religion-wise, 82.6% of males and 97% of females were Hindus and the rest were of Muslim faith (*P* = 0.037). Although 30.4% of males were educated up to the secondary level, the majority (33.3%) of the female cases were illiterate (*P* = 0.018). Illiterate means they cannot read nor write and can not sign their names. In males, most common were students (26.1%) while the majority of the females (83.3%) were housewives (*P* < 0.001). Mean (standard deviation) age for males was 30.47 (10.45) years and that for females was 27.53 (14.73) years.

### 3.2. Clinical Profile

Findings from clinical assessments are summarised in Table 1.

#### 3.2.1. Intention to Die

Most of the subjects (69.7%) were uncertain about their “intention to die” from the self-harm act. Although females showed greater preponderance in their uncertainty, but the observed difference was not statistically significant.

#### 3.2.2. DSH Method

Use of poison was by far the commonest method in both sexes, with 100% of females and 82.6% of males using it. Hanging (17.4%) was seen only among males. This difference was significant. The majority of subjects, 63.2% males and 66.7% females, used commonly accessible agrochemical pesticides. The sex difference in use of pesticides and household chemicals was not significant. Drugs were used by a few men and indigenous poisonous plants by a few women. The type of poison used is summarised in Table 2. 

#### 3.2.3. Location

Maximum number of cases (89.9%) had home as venue for DSH. Home was significantly chosen as venue by females (93.9%) compared to males (78.3%). 

#### 3.2.4. Help Seeking

Most of the cases (97.8%) did not seek any help. Help seeking was higher among males (4.3%) compared to females (1.5%). This difference is not significant statistically.

#### 3.2.5. Past History of DSH

16.9% of cases had past history of DSH, which was more common in males (26.1%) compared to females (13.6%), although this difference was not significant.

Table 3 shows the causes for DSH according to gender. Conflict with spouse was by far the commonest cause in both the sexes (with some female predominance) followed by conflict with guardians (more common in males), but this difference was not significant. Other causes included tension with in-laws over infertility, dowry, extramarital affair of spouse, chronic illness, and failed love affairs. Surprisingly, economic distress prompted the DSH event in only 2 male subjects.

### 3.3. DSM-IV Psychiatric Diagnosis

As Table 4 shows, more female cases (65.2%) were free from both Axis I and Axis II conditions compared to males (47.8%), a difference not statistically significant. Axis I disorder without presence of borderline personality disorder (BPD) was seen more among males (34.8%) compared to females (24.2%). Again, this observed difference was not significant. Considering the diagnostic distribution, the affective disorder spectrum covered the majority of the psychiatric diagnoses: 30.4% in males and 24.2% in females. Individually, major depressive disorder (MDD) was the commonest diagnosis (14.6% overall), being slightly more common in males (17.4%) than females (13.6%). Interestingly 47.8% males (11) and 65.2% females (43) did not fulfil any diagnostic criteria for psychiatric illness.

### 3.4. EMIC Interviews

Among the subset of 54 cases without any psychiatric diagnosis, EMIC interview revealed different psychosocial causes as the underlying distress as well as triggering factors for DSH (Table 5). Among males, the most frequently implicated causes were conflict with parents (36.3%) followed by marital discord (27.3%). In females, the leading causes were marital discord (37.2%) followed by domestic violence (20.9%). These differences were statistically not significant. 

## 4. Discussion

The present research in a primary care setting of the Sundarban region reveals some interesting findings about sociodemographic variables and psychiatric morbidity of self-harm behaviour. It reveals that in both sexes suicidal behaviour is more frequent among individuals in their 30s, corroborating observations of earlier Indian studies [28, 29]. It is interesting to note that female subjects were found to be younger than males. In the present series, female preponderance over males is noted. Though this finding is in agreement with some earlier studies [6, 20, 30], the opposite has also been reported from the Indian context [28, 31]. Low educational attainment (30.3% being illiterate) among the cases was a significant finding and this agrees with other studies [4, 30]. Occupation-wise, most were doing “nil (housewife)” among females whereas students dominated among males in the present study. The high percentage of housewives (83.3%) in the present sample is definitely far above the usual average of regional married female population (about 44%). The high frequency of self-harm attempt among housewives and students has been reported from Himachal Pradesh, India [6].

DSH attempt was found more in married individuals, more common in females [5, 23]. Experience gained through focus group discussions (FGD) with housewives in Sundarban region evinced the stressful life of married women, like that in most Indian villages. Married women are exposed to a variety of psychosocial stresses, ranging from dowry demand and torture, hard physical labour (both domestic and in the field), lack of adequate privacy and recreational facilities, to, as in many cases, the physical and mental humiliation and torture by alcoholic husbands or in-laws. Social support is inadequate. The authors believe that these factors have a strong role in enhancing psychodynamic vulnerability for DSH among married women.

Most of the cases here were uncertain about their intention of dying from the self-harm act. Though not significant, women showed proportionately greater uncertainty about their intention. This finding is in concurrence with Hawton's study [32], which suggested that in cases of women there is less motivation for suicide. Detailed interview also revealed that, especially among the women, self-harm behaviour is either sudden, impulsive, or an attempt to escape from a stressful situation and is often used for seeking attention rather than ending life. In some cases it resembles hysterical conversion reaction to interpersonal stress. Another point of clinical interest here is that “all attempted suicide” cases at the BPHC need police reporting as according to the Indian Penal Code this is an offense. Many cases in the EMIC interview said that in order to avoid legal complication (if say yes) and social humiliation or stigma (if say no—*Jhuki*) (Jhukimara. Deliberate self-harm attempt without definite desire to die is locally known as “Jukhimara” or simply “Jukhi.” The literal meaning of the term is “taking risk” (of life). The incumbent in most cases either wants to communicate his/her sufferings as an alarm or wants to achieve something (ranging from social punishment to in-laws, diminution of parental pressure for studies, etc.). Socially, *Jukhi* is seen as a bad character, and often such history negatively influences marriage negotiation for girls.) they preferred to opt for “uncertain” response. This was probably the reason for which nobody responded to the “no” option. This psychosocial dynamics need further study.

Poisoning is regarded as a leading cause of death in rural and agricultural areas across the world [33]. Irrespective of gender, poisoning was the most common mode (95.5%) of DSH attempts in this study. Studies from rural background have also reported poisoning as the commonest method for DSH [34, 35]. The field observation revealed that recent agricultural innovations have prompted local farmers to harvest two or three crops in a year. They use (or overuse) different types of lethal pesticides, mainly organophosphorus compounds, to safeguard the crops. Because of their ignorance and carelessness in storage of these pesticides, lethal poisons are easily available and accessible to any member, even toddlers, in the household. Also, there is neither any control on sale and purchase of pesticides in the entire region nor any safety information dissemination to the farmers. To cite a few examples, about 98% of pesticide shops in the area are unlicensed; pesticides are available even in grocery shops; any person, be it an excited housewife or a young adolescent, can purchase any amount and type of pesticide without hindrance from these shops. A study in the Sundarban region reported similar observations [36]. Recent agricultural innovations (chemical fertilizers and pesticides) in the developing countries have been replacing the traditional mode of DSH by hanging or drowning by pesticide ingestions [37].

Home was the most preferred (89.9%) location for DSH acts in the present study. Women made higher proportion of attempts at home than men. This finding is in concurrence with other studies [38, 39], and the preference for home needs further research. One possible reason here may be that many of the present cases did not have the intention to die, so that they considered home a safe place to be discovered and rescued quickly. 

Marital disharmony and family conflict were found to be the major triggers for self-harm attempt in the present sample. Conflict with spouse was by far the commonest cause in both sexes followed by quarrel with guardians and in-laws. The most common causes of conflict were domestic violence related with husband's alcoholic, abusive behaviour [40] or his extramarital affairs or dowry or household work related with conflicts with in-laws. This highlights the need for clinical attention and counselling of the cases with familial maladjustment because the cumulative effects of emotional stresses increase the vulnerability to DSH. In many of the cases interpersonal maladjustment precipitates quarrel and that acted as trigger for the impulsive self-harm attempt. Quarrel as a significant risk factor was also noted in a study from Taiwan [41]. Recent studies, from both developing [42, 43] and developed countries [44, 45] also recognised increasingly that DSH is a common response to acute emotional distress.

The status of psychiatric diagnosis merits close attention. It is interesting to note that designated psychiatric disorders among the DSH cases follow a diagnostic distribution similar to the urban Indian setting [46]. The major depressive disorder was the commonest psychiatric diagnosis irrespective of gender. This finding highlights the urgent need for diagnosis and treatment of this disorder at the primary care level. The second most common diagnosis was adjustment disorder (6.7%). Despite being a clinical diagnosis, it receives very little attention in medical practice, especially in primary care. Bhatia et al. [47] in their study of DSH attempters from Delhi found that adjustment disorder with depression was the commonest diagnosis. Familial maladjustment is largely viewed by health staff as a “personal problem” and thus escapes proper clinical attention. Careful scrutiny is needed to identify this situation for proper psychosocial intervention. The situation is similar regarding the diagnosis of borderline personality disorder. Either the DSH cases in this part of the world are not having high rate of comorbid BPD or there is a lack of clinical awareness in this diagnosis. This area needs further research. Professional awareness about Axis II disorders in developing countries is not like the western world, and there is a need for proper training for more precise clinical decision making and thus suitable management protocol for these cases. Psychiatric morbidity is a very important risk factor in DSH [48] and there is no diagnostic definition of DSH in DSM (IV) or ICD 10 [49], and thus more professional precision is required for proper diagnostic identification of vulnerability and subsequent management.

Contrary to the general belief, most of the cases bore “no psychiatric diagnosis” (60.7%). More female cases were free from both Axis I and Axis II disorders of DSM-IV diagnosis compared to males. This finding of absence of designated psychiatric illness associated with self-harm behaviour is in agreement with some earlier studies [30, 31, 50]. It invites serious public health attention so far as DSH prevention activities are concerned [51]. Most probably, these cases reflect strong, momentary, impulsive self-harm behaviour without any background of psychiatric morbidity. Impulsivity may play a greater role in DSH than previously thought, and associated easy availability of means of self-harm has considerable impact on the decision to act [52]. It appears from the EMIC interviews that in many of the cases the self-harm behaviour was primarily of attention-seeking nature (e.g., conscious about the small amount and nonlethality of the pesticides, attempted when somebody is at home, believed that this attempt will invite focus in resolving his/her ongoing stress like relational problem with husband or mother-in-law, pressure of study, or reconciliation of broken love relation). So, from the point of community-based psychosocial intervention, this area needs careful consideration. Interestingly, none of the cases had any history of regular alcohol drinking and none attempted DSH under the influence of alcohol. This is a very striking observation and merits exploration through a larger study.

Thus, we can conclude from the present study that younger age (around 30 years), female sex, low educational level, married status, and intrafamilial conflicts (with spouse or guardian) are the significant risk factors associated with DSH behaviour in the Sundarban region. Pesticide poisoning was the most common method of DSH, reflecting a strong association between impulsive behaviour and ready availability of pesticides in the region. Preventive activity should highlight issues like timely psychosocial intervention to alleviate familial maladjustment in order to reduce the mortality and morbidity. Official regulation of sale and purchase of pesticides and education of farmers about proper use and safe storage of pesticides need to be addressed in the preventive activity in this agrarian community. Designated psychiatric illness was not found in the majority of the cases. This resembles the suicide pattern in China [53], where the social dimensions of suicide and correlation with social problems have been emphasised. More than three decades ago, Hankoff [54] also documented that serious psychopathology probably accounts for a very small proportion of attempted suicides. This view strongly relates to the more recent findings of [55] that reported that designated psychiatric illnesses are less seen in DSH attempt cases than cases of completed suicide. It is to be reiterated that subjects with no designated psychiatric disorders, including adjustment disorder, require more psychosocial management than pharmacotherapy, and herein lays the importance of public education, community-based psychosocial intervention, and professional awareness [56, 57]. A community DSH prevention program should include these components along with provisions for treatment and management of acute poisoning and psychiatric diseases [58, 59].

This research also demonstrates the need for further study in different settings of the relative explanatory power of psychopathological and social determinants of suicidal behaviour. Highlighting the importance of social determinants of DSH in India heightens interest in, rather than answering, the question of whether the emphasis on social determinants represents a true crosscultural difference or the need to expand the scope of mental health research and activity in Europe and North America. In either case, findings clearly indicate the importance of innovating new models in planning for the mental health of populations. The study shows the interest of community mental health in promoting social support and positive mental health is likely to be especially pertinent for suicide prevention.

Certain limitations of the study need to be mentioned here. As DSH attempt is a punishable offence and carries strong social stigma, detailed information collection about the DSH act was difficult. Many of the cases showed no interest for the detailed clinical interview. Moreover, interference by family members was a problem during the interview in some of the cases. In a few instances, interviews were stopped half way because of the objections raised by family members who probably apprehended legal trouble. Incomplete interviews were more with female subjects. With a limited sample size, the study findings remain somewhat tentative.

## Figures and Tables

**Table 1:** Summary of issues related to the deliberate self-harm act in the study population.

Clinical variable	Male (*n* = 23)	Female (*n* = 66)	Overall (*n* = 89)	Male : female comparison *P* value
Intent to die				
Affirms	9 [39.1]	18 [27.3]	27 [30.3]	
Uncertain	14 [60.9]	48 [72.7]	62 [69.7]	0.303
Method				
Poisoning	19 [82.6]	66 [100.0]	85 [95.5]	
Hanging	4 [17.4]	—	4 [4.5]	0.004
Location				
Home	18 [78.3]	62 [93.9]	80 [89.9]	
Outside	5 [21.7]	4 [6.1]	9 [10.1]	0.046
Help seeking				
Sought help	1 [4.3]	1 [1.5]	2 [2.2]	
Did not seek help	22 [95.7]	65 [98.5]	87 [97.8]	0.452
Past history of DSH attempt				
Yes	6 [26.1]	9 [13.6]	15 [16.9]	
No	17 [73.9]	57 [86.4]	74 [83.1]	0.201

Figures in parentheses indicate percentages.

**Table 2:** Profile of the poisons used in the deliberate self-harm acts.

Poison	Male (*n* = 19)	Female (*n* = 66)	Total (*n* = 85)
Agrochemical pesticides	12 [63.2]	44 [66.7]	56 [65.9]
Household chemicals	5 [26.3]	17 [25.8]	22 [25.9]
Indigenous poisons	—	5 [7.5]	5 [5.9]
Medicine	2 [10.5]	—	2 [2.4]

The pesticides used were mostly organophosphorus compounds; the household chemicals used included Dettol (a chloroxylenol disinfectant), kerosene, caustic washing soda; the indigenous poisons included seeds of Yellow Oleander; while the drugs used were diazepam and antacid tablets.

**Table 3:** Gender and cause of DSH (at first clinical contact).

Psychosocial distress	Male (*n* = 23)	Female (*n* = 66)	Overall (*n* = 89)
Marital conflict	8 [34.8]	30 [45.5]*	38 [42.7]
Conflict with parents/guardians	11 [47.8]	15 [22.7]	26 [29.2]
Conflict with in-laws	—	10 [15.2]	10 [11.2]
Dowry related	1 [4.3]	5 [7.6]	6 [6.7]
Chronic illness	1 [4.3]	2 [3.0]	3 [3.4]
Broken love affair	—	2 [3.0]	2 [2.2]
Economic distress	2 [8.7]	—	2 [2.2]
Failure in examination	—	2 [3.0]	2 [2.2]

Figures in parentheses indicate percentages.

*17 (56.7%) cases related with husband's alcohol use.

**Table 4:** Diagnostic distribution of the cases.

Psychosocial distress	Male (*n* = 23)	Female (*n* = 66)	Overall (*n* = 89)
Axis I disorder			
MDD	4 [17.4]	9 [13.6]	13 [14.6]
Dysthymia	1 [4.3]	1 [1.5]	2 [2.2]
Bipolar II—hypomanic episode	1 [4.3]	—	1 [1.1]
MDD—general medical condition	—	1 [1.5]	1 [1.1]
MDD with BPD	1 [4.3]	4 [6.1]	5 [5.6]
Depressive disorder NOS	—	1 [1.5]	1 [1.1]
Affective disorder spectrum	**7 [30.4]**	**16 [24.2]**	**23 [25.8]**
Schizophrenia	2 [8.7]	2 [3.0]	4 [4.5]
Adjustment disorder	2 [8.7]	4 [6.1]	6 [6.7]
Axis II disorder			
BPD	1 [4.3]	1 [1.5]	2 [2.2]
No psychiatric diagnosis	11 [47.8]	43 [65.2]	54 [60.7]

Figures in parentheses indicate percentages.

Abbreviations: BPD: borderline personality disorder; MDD: major depressive disorder; NOS: not otherwise specified.

**Table 5:** Psychosocial causes of DSH among non-psychiatric cases.

Psychosocial distress	Male (*n* = 11)	Female (*n* = 43)	Overall (*n* = 54)
Marital discord	3 [27.3]	16 [37.2]*	19 [35.2]
Domestic violence	1 [9.1]	9 [20.9]**	10 [18.5]
Conflict with parents	4 [36.3]	4 [9.3]	8 [14.8]
Family discord	—	7 [16.3]	7 [12.9]
Social shame	1 [9.1]	2 [4.6]	3 [5.6]
Threat of rejection	1 [9.1]	1 [2.3]	2 [3.7]
Examination failure	1 [9.1]	1 [2.3]	2 [3.7]
Mental shock	—	2 [4.6]	2 [3.7]
Economic stress	—	1 [2.3]	1 [1.8]

Figures in parentheses indicate percentages.

*4 (25%) cases related with husband's alcohol use.

**5 (55.5%) cases related with husband's alcohol use.
